# Application of Machine Learning in Postural Control Kinematics for the Diagnosis of Alzheimer's Disease

**DOI:** 10.1155/2016/3891253

**Published:** 2016-12-18

**Authors:** Luís Costa, Miguel F. Gago, Darya Yelshyna, Jaime Ferreira, Hélder David Silva, Luís Rocha, Nuno Sousa, Estela Bicho

**Affiliations:** ^1^Algoritmi Center, Department of Industrial Electronics, School of Engineering, University of Minho, Braga, Portugal; ^2^Neurology Department, Hospital da Senhora da Oliveira, Guimarães, Portugal; ^3^Life and Health Sciences Research Institute (ICVS), School of Health Sciences, University of Minho, Braga, Portugal; ^4^ICVS-3B's-PT Government Associate Laboratory, Braga, Portugal

## Abstract

The use of wearable devices to study gait and postural control is a growing field on neurodegenerative disorders such as Alzheimer's disease (AD). In this paper, we investigate if machine-learning classifiers offer the discriminative power for the diagnosis of AD based on postural control kinematics. We compared Support Vector Machines (SVMs), Multiple Layer Perceptrons (MLPs), Radial Basis Function Neural Networks (RBNs), and Deep Belief Networks (DBNs) on 72 participants (36 AD patients and 36 healthy subjects) exposed to seven increasingly difficult postural tasks. The decisional space was composed of 18 kinematic variables (adjusted for age, education, height, and weight), with or without neuropsychological evaluation (Montreal cognitive assessment (MoCA) score), top ranked in an error incremental analysis. Classification results were based on threefold cross validation of 50 independent and randomized runs sets: training (50%), test (40%), and validation (10%). Having a decisional space relying solely on postural kinematics, accuracy of AD diagnosis ranged from 71.7 to 86.1%. Adding the MoCA variable, the accuracy ranged between 91 and 96.6%. MLP classifier achieved top performance in both decisional spaces. Having comprehended the interdynamic interaction between postural stability and cognitive performance, our results endorse machine-learning models as a useful tool for computer-aided diagnosis of AD based on postural control kinematics.

## 1. Introduction

Around 30% of the people aged more than 65, living in the community, and more than 50% of those living in residential care facilities or nursing homes fall every year. Moreover, about half of those who fall do so repeatedly [1]. With the increase in the elderly population, the number of falls in this group has been rising constituting an important public health problem [2]. Postural instability, characterized by excessive and uncontrolled sway, degrades with ageing and is a risk factor for the occurrence of falls, especially in neurodegenerative diseases, such as Alzheimer's disease (AD) [3]. AD is a neurodegenerative cortical disorder that besides memory deficits also displays disturbances of posture and gait, which triggers more serious falls compared to nondemented elderly people. In that regard, diagnostic tools that allow an early and noninvasive detection of AD pathology are highly required.

To this end, many researchers have devoted their efforts to find appropriate data/features and have applied different machine-learning methods for computer-aided diagnosis of AD. Most of the works reported in the literature make use of Support Vector Machines (SVMs) and Artificial Neural Networks (ANNs), such as Multiple Layer Perceptrons (MLPs), Radial Basis Function Networks (RBNs), and Deep Belief Networks (DBNs). We provide a brief review next in this context.

SVMs are a particular type of supervised machine-learning method that classifies data points by maximizing the margin between classes in a high-dimensional space [4]. They are the most widely used classifiers and have shown promising results on problems of pattern recognition in neurology and psychiatry diseases [5], including detection of AD based on electrical brain activity Electroencephalography (EEG) [6], neuroimaging data from Magnetic Resonance Imaging (MRI), and Positron Emission Tomography (PET) brain images [7–10]. Several works have applied MLPs in the diagnosis of AD, combining different variables such as demographic, neurological, and psychiatric evaluation, neuropsychological tests, and even more complex clinical diagnostic tools (e.g., neuropathology, EEG, and MRI/PET brain imaging), where hundreds of variables of recorded data are potentially clinically relevant on one single patient [7, 11, 12]. RBNs have successfully been applied to the discrimination of plasma signalling proteins for the prediction of this disease [13] and classification of MRI features of AD [7]. DBNs are a recent machine-learning model that is exhibiting performance records on classification accuracy also on medical fields such as AD, based on MRI/PET neuroimaging data [14, 15].

The survey of the above literature shows that the majority of the studies have relied on neuroimaging data from MRI and/or PET images, which though widely available, are relatively expensive. In contrast, inertial measurement units (IMUs), with integrated accelerometers and gyroscopes, are inexpensive and small fully portable devices, opening a new field of research on AD. In fact, IMUs have been used to portrait different postural kinematic profiles in AD, including a higher risk of falling [16]. These devices are independent of inclination in space, having proved to be equivalent to force platforms in the evaluation of the center of mass (COM) kinematics. However, although hundreds of kinematic parameters have been used to represent postural body sway [17], which parameters provide the most relevant information about normal postural control and which kinematic parameters better identify neurodegenerative diseases such as AD are still yet undetermined. We advocate that a complementary tool that makes use of kinematic postural data for the diagnosis of AD would be extremely helpful and valuable for clinicians.

To the best of our knowledge, the use of machine-learning classifiers for the diagnosis of AD based on kinematic postural sway data has not yet been investigated. With this in mind, our study has two main goals. First, to validate the feasibility of the application of machine-learning models in the diagnosis of AD based on postural kinematic data, collected on different and increasingly difficult postural balance tasks. Second, to compare different classifier models—SVM, MLP, RBN, and DBN—with respect to their discriminative performance.

The remainder of the paper is structured as follows. In Section 2 we explain the materials and methodology used for collecting the data, feature reduction, and implementation of the three dataset models, subsequently used for training, testing, and comparing the different classifiers models.  Section 3 gives a brief description on how we implemented the classifiers' models.  Section 4 presents results of performance for the different classifiers in the different dataset models. In Section 5 a detailed discussion is made such that, in Section 6, some conclusions can be drawn about the potential use of the tested classifiers in future automatic diagnostic tools for AD based on kinematic postural sway data.

## 2. Materials and Methodology for Data Collection

### 2.1. Study Population

The study population was recruited from our hospital outpatient neurology department. Patients with probable AD, according to Diagnostic and Statistical Manual of Mental Disorders- IV (DSM-IV) and National Institute of Neurological and Communicative Disorders and Stroke/Alzheimer's Disease and Related Disorders Association (NINCDS/ADRDA) criteria [18], on a stage of 1 on the Clinical Dementia Rating Scale, were consecutively recruited for the study. The control group included age-matched caregivers of patients that had no history of falls or of neurological or psychiatric disease. Patients or controls were excluded if there was a history of orthopedic, musculoskeletal, vestibular disorder, or alcohol abuse. Demographic, anthropometric, and MoCA data, normalized to the Portuguese population [19], were collected in both groups. Local hospital ethics committee approved the protocol of the study, submitted by ICVS/UM and Center Algoritmi/UM. Written consent was obtained from all subjects or their guardians.

We included 36 AD patients (24 females/12 males, with a mean age of 76 ± 7 years) and 36 healthy controls (15 females/21 males, with a mean age of 70 ± 8 years) (AD versus C, *p* = 0.003). Concerning demographic and anthropometric data, the two groups displayed the following: education (AD: 1 ± 0.58; control: 2 ± 1.19; *p* = 0.008); MoCA (AD: 11 ± 5.10; control: 25 ± 3.87; *p* < 0.001); weight (kg) (AD: 65.60 ± 10.28; control: 75.24 ± 12.11; *p* = 0.001); height (m) (AD: 1.54 ± 0.08; control: 1.63 ± 0.106; *p* < 0.001); body mass index (kg/m^2^) (AD: 27.58 ± 4.12; control: 28.24 ± 3.79; *p* = 0.44). These significant differences between the two groups justified the adjustment of the kinematic variables to age, education, height, and weight (please see below).

### 2.2. Kinematic Acquisition and Assessment System

Five kinetic sensing modules harboring 8051 microprocessor embedded in CC2530* Texas Instrument* SoC (System on Chip) [20] and an inertial measurement unit MPU6000 (triaxial accelerometer and gyroscope), operating with a sample rate frequency of 113 Hz on SD card, were attached to five body segments: trunk (on the COM, located at 55% of a person's height [21]), both legs (middle of ankle-knee), and both thighs (middle of knee-iliac crest) by Velcro bands. One of the normal human mechanisms of maintaining balance is to vary the height of the COM. Therefore, final kinematic information derived from the IMU on the COM was constantly adjusted to the angle and length of the IMU located on the thigh and shank. A more detailed description of our methodology and mathematical formulas for kinematic acquisition procedure can be consulted at [16].

### 2.3. Clinical Postural Tasks

**Figure 1:**
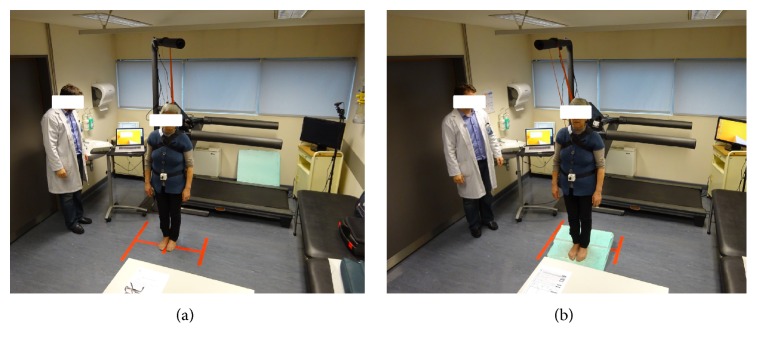
Representation of a patient, wearing the safety trunk belt, with the IMU placed on the center of mass (55% height), while performing the tasks: eyes closed with feet together on flat surface (a) and on frontwards platform (b) (other tasks were performed, as further detailed on the Materials and Methodology for Data Collection).

Subjects were instructed to perform seven different postural tasks with increasing stability stress: normal stance: standing with the medial aspects of the feet touching each other with eyes open (EO) and eyes closed (EC), and standing with the medial aspects of the feet touching each other with EO and EC on a ramp with 15 degrees' inclination in a backwards position (EOBP, ECBP) and frontwards position (EOFP, ECFP) [22]. A representation of a patient, wearing the safety trunk belt, with the IMU placed on the center of mass while performing the tasks mentioned, can be seen in Figure 1. Tasks with kinematic capture were performed for 30 seconds [23], with subjects standing quiet, their arms hanging at their sides, and their head in a normal forward-looking position to a visual eye target height approximately 2 meters away. Balance is a complex process of coordination of multiple body systems—including the vestibular, auditory, visual, motor, and higher level premotor systems—that generates appropriate synergic postural muscle movements of the head, eye, trunk, and limbs to maintain posture [24]. This is achieved by sustaining, achieving, or restoring the body COM relative to the base of support or, more generally, within the limits of stability with minimal sway [25]. Visual suppression makes the human body more dependent on vestibular and proprioceptive systems, consequently increasing sway [26]. On an inclined or tilting support surface, postural control is mainly achieved with the help of visual, vestibular, and proprioceptive afferents. The investigation of postural stability under dynamic conditions, either continuous or predictable perturbations of the supporting platform, has been used to study anticipatory adjustments and sensory feedback [24]. This was the rationale in our study to use different and increasing difficulty postural stability tasks, changing kinematic variables, in order to obtain more information for machine-learning analysis and discrimination between patients and healthy subjects.

### 2.4. Kinematic Collected Variables

**Figure 2:**
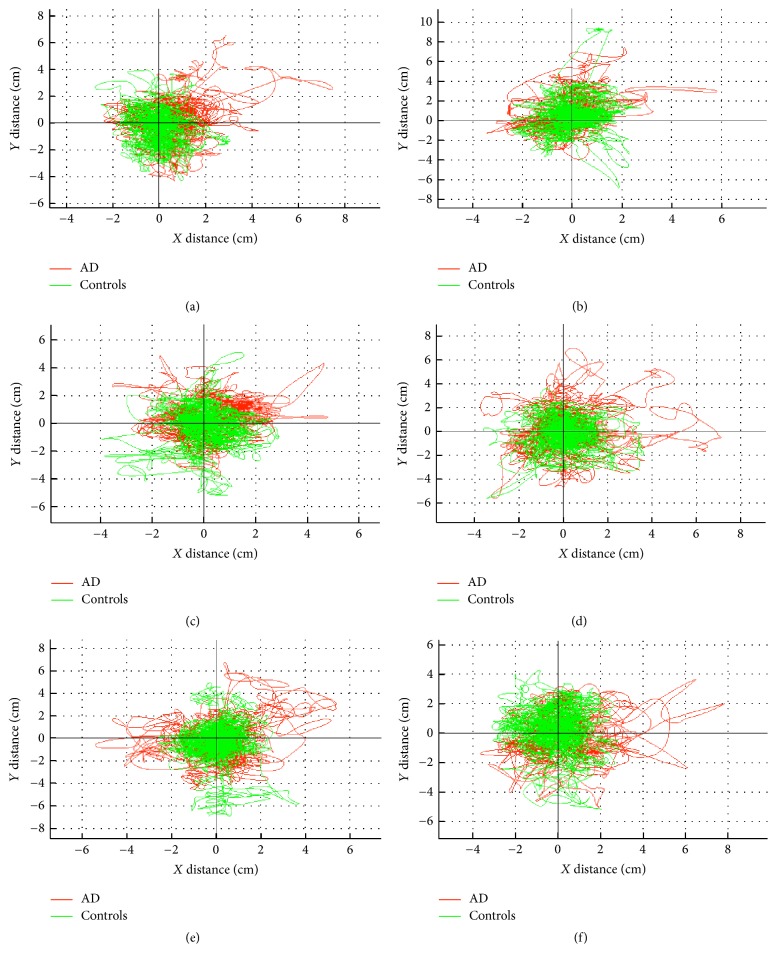
Representation of the substantial overlap on the different kinematic postural features, on an orthogonal projection, between the two groups and the different tasks, (a) EO, (b) EOFW, (c) EOBW, (d) EC, (e) ECFW, and (f) ECBW, with increasing difficulty of postural stability.

We focused on demographic and biometric data (age, weight, height, and body mass index) and kinematic parameters (extracted from the IMU placed at the COM) that emerged from a systematic review as predictors of falls among older people and AD patients [26–30]. Kinematic parameters are as follows: total displacement on the transverse plane (cm); maximal displacement (cm) with respect to the origin; mean distance (cm) with respect to origin on transverse plane; dispersion radius (average distances relative to average point); maximal and mean linear velocity (cm/s); positioning (cm) on *x*-axis (maximal, mean, and range) and *y*-axis (maximal, mean, and range); roll angle (degrees) (maximal, minimum, and mean); and pitch angle (degrees) (maximal, minimum and mean). These 18 kinematic measurements, captured on each task, were further averaged summarizing the patient's behavior throughout the seven different postural tasks. The overlap between the different kinematic postural features between the two groups and the different tasks can be seen in Figure 2.

### 2.5. Feature Extraction and Statistical Significance

There is still little information about the value of each singular kinematic variable, and even less information exists on how these variables interact among themselves during postural balance. During data collection on the different postural tasks, there is substantial overlap of kinematic information, even if we only consider one particular variable, like displacement on the *x* and *y*-axis (Figure 2). Therefore, the objective of the feature extraction process is to assess which of the kinematic variables are statistically significant features that contribute to an accurate classification of AD patients.

As in [31], all kinematic variables were adjusted for age, education, height, and weight (as these were found to be significant factors, with a significance value of 0.05, using the Mann–Whitney* U* test and Chi-Square test):

(1) Ka=Kua−GageKsAge−KmAge−GwghtKsWght−KmWght−GhghtKsHght−KmHght−GEduKsEdu−KmEdu,

where *K*_a_ is the adjusted kinematic feature; *K*_ua_ is the unadjusted kinematic feature; *K*_sAge_,  *K*_sWght_,  *K*_sHght_, and *K*_sEdu_ are the subject's age, weight, height, and years of education, respectively; *K*_mAge_, *K*_mWght_, *K*_mHght_ and *K*_mEdu_ are the corresponding means for all subjects. The gradients *G*_age_, *G*_wght_, *G*_hght_, and *G*_Edu_ are the slopes of a region specific regression line against subject age, weight, height, and education of all participants. This process of adjustment guarantees that the regression is not influenced by the classification of each variable in particular.

Data is then preprocessed by a min–max normalization method (see, e.g., [32]):

(2) x′=x−min⁡g∗new_max⁡g−new_min⁡gmax⁡g−min⁡g+new_min⁡g⁡

and that, in our case, transformed data into a range of values between −1 and 1. Thus, new_max⁡(*g*) and new_min⁡(*g*)⁡ were set to 1 and −1, respectively, and max⁡(*g*) and min⁡(*g*)⁡ are the maximum and minimum values of the attribute, respectively. Afterwards, a nonparametric statistical analysis (Mann–Whitney* U* test) is implemented to determine the rank and significance, of each variable, in the classification outcome of the two groups, following one branch considering solely the 18 kinematic variables, and the second branch including the MoCA score as to form a 19-variable vector for each subject.

### 2.6. Variable Selection Using Error Incremental Analysis

**Figure 3:**
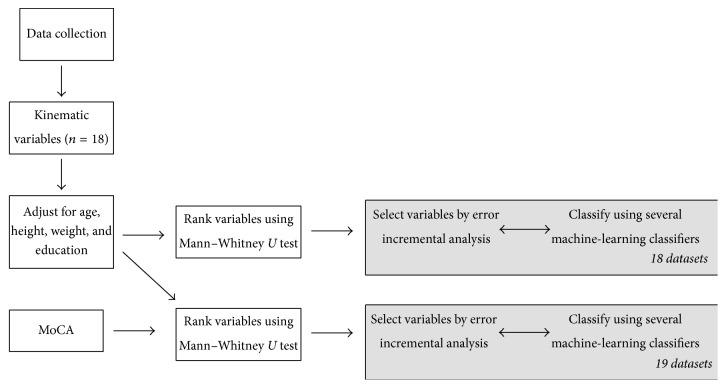
Methodological flowchart of the classification approach.

As per [10], the ranking of the statistically significant variables provides an insight on the discriminative power of each variable for each classifier. Selecting the optimal number of top-ranked variables can be considered a dimensionality reduction problem which is performed using error incremental analysis: starting from the top-ranked variable and incrementally adding the next best ranked variable until all significant variables are included. The methodology followed in this study is presented in Figure 3.

## 3. Machine-Learning Classifiers

The selection of the best classifier for diagnosis is an open problem. In addition, the advantage of using multiple classification models over a single model has been suggested [32]. Hence, we compare four different classifier models: SVM, MLP, RBN, and DBN. With the purpose of facilitating and streamlining the work, we developed a custom-made software application on MATLAB® (version R2014a), which implements an automatic grid-search (i.e., automatically and systematically tests different configurations and performance of the different machine-learning models).

All the experiments were based on a threefold cross validation, meaning that the subjects were divided into three sets: training (50%), test (40%), and validation (10%) [33]. To limit the potential data partitioning error induced by random data assignment and cross validation, the same experiment was repeated 50 times and the average performance was recorded. We opted for an output layer composed of two neurons, one representing AD patients and the other healthy/control subjects, as this model would better replicate clinical practice.

### 3.1. Support Vector Machines (SVMs)

The learning mechanism of a SVM considers distinct classes of examples as being divided by geometrical surfaces, separating hyperplanes, whose optimal behavior is determined by an extension of the method of Lagrange multipliers. The support vector classifier chooses the classifier that separates the classes with maximal margin [34]. Our implementation of SVM follows the MATLAB Documentation and [35].

We provide a brief description, but for more detailed information refer to the respective references.

Let us assume that the dataset is of the form

(3) $$\begin{array}{c} D={\left\{\;x_{k},o_{k}\;\right\}}_{k=1}^{k=m}, \end{array}$$

where **x**_*k*_ ∈ *ℝ* ^*m*^ is the *k*th input vector of dimension *m* and *o*_*k*_ is the corresponding binary category, *o*_*k*_ ∈ {−1,1}.

The equation that defines the hyperplane is

(4) $$\begin{array}{c} \langle \;v,x_{k}\;\rangle +b=0, \end{array}$$

where **v** ∈ *ℝ* ^*m*^ is the vector normal to the hyperplane, 〈·〉 represents the inner product, and* b*, a real number, is the bias.

In order to define the best separating hyperplane one needs to find **v** and *b* that minimize ‖**v**‖ subject to

(5) $$\begin{array}{c} o_{k}\left(\;\langle \;v,x_{k}\;\rangle +b\;\right)\geq 1. \end{array}$$

In order to simplify the math, the problem is usually given as the equivalent of minimizing 〈*v*, **v**〉/2.

Once the optimal **v** and *b* are found, one can classify a given vector, **z**, as follows:

(6) $$\begin{array}{c} y_{z}=\langle \;v,z\;\rangle +b, \end{array}$$

where *y*_*z*_ is the binary category in which **z** is inserted. This is considered to be the primal form of the classification problem.

In order to attain the dual form of the classification problem, one needs to take the Lagrange multipliers, *α*_*k*_, multiplied by each constraint and subtract from the objective function:

(7) $$\begin{array}{c} L_{p}=\frac{1}{2}\langle \;v,v\;\rangle -\overset{N}{\underset{k=1}{\sum }}\alpha_{k}\left(\;o_{k}\left(\;\langle \;v,x_{k}\;\rangle +b\;\right)-1\;\right), \end{array}$$

where *N* is the size of the training data.

The first-order optimal conditions of the primal problem are obtained by taking partial derivatives of *L*_*p*_ with respect to the primal variables and then setting them to zero:

(8) $$\begin{array}{c} \frac{\partial L_{p}}{\partial v}=0⟶v=\overset{N}{\underset{k=1}{\sum }}\alpha_{k}o_{k}x_{k}. \end{array}$$

The dual form of the classification problem is obtained as follows:

(9) $$\begin{array}{c} L_{D}=\overset{N}{\underset{k=1}{\sum }}\alpha_{k}-\frac{1}{2}\overset{N}{\underset{k=1}{\sum }}\overset{N}{\underset{\;j=1}{\sum }}\alpha_{k}\alpha_{j}o_{k}o_{j}\langle \;x_{k},x_{j}\;\rangle \end{array}$$

subject to constraints

(10) $$\begin{array}{c} \overset{N}{\underset{j=1}{\sum }}\alpha_{k}o_{k}=0,\;0\leq \alpha_{k}\leq c, \end{array}$$

where *c* is considered a constraint value that keeps the allowable values of the Lagrange multipliers, *α*_*k*_, in a bounded region.

Some classification problems cannot be solved with the linear methods explained above because they do not have a simple hyperplane as a separating criterion. For those problems, one needs to use a nonlinear transformation, and that is achievable through the use of kernels [34].

Assuming *F* is a high-dimensional feature space and *φ* is a function that maps **x**_*k*_ to *F*, the kernel has the following form:

(11) $$\begin{array}{c} K\left(\;x_{k},x_{j}\;\right)=\langle \;\varphi \left(\;x_{k}\;\right),\varphi \left(\;x_{j}\;\right)\;\rangle . \end{array}$$

In our implementation we used the Gaussian kernel function defined as follows:

(12) $$\begin{array}{c} K\left(\;x_{k},x_{j}\;\right)=\langle \;\varphi \left(\;x_{k}\;\right),\varphi \left(\;x_{j}\;\right)\;\rangle =e^{-\langle \left(x_{k}-x_{j}\right),\left(x_{k}-x_{j}\right)\rangle /{2\sigma }^{2}}, \end{array}$$

where *σ* is a positive number.

Applying the kernel to the dual form of the classification problem, one obtains

(13) $$\begin{array}{c} L_{D}=\overset{N}{\underset{k=1}{\sum }}\alpha_{k}-\frac{1}{2}\overset{N}{\underset{k=1}{\sum }}\overset{N}{\underset{\;j=1}{\sum }}\alpha_{k}\alpha_{j}o_{k}o_{j}K\left(\;x_{k},x_{j}\;\right) \end{array}$$

subject to constraints

(14) $$\begin{array}{c} \overset{N}{\underset{j=1}{\sum }}\alpha_{k}o_{k}=0,\;0\leq \alpha_{k}\leq c. \end{array}$$

### 3.2. Multiple Layer Perceptrons (MLPs)

We have previously detailed our MLP model [36], where computation of the output of neuron *y*_*j*_ was based on the following:

(15) yj=g∑i=0Nwjilnyil−1n,gvj=11+e−vj,

where *y*_*i*_^(*l* − 1)^(*n*) is the output of neuron *i* in the previous layer *l* − 1 at iteration *n* and *w*_*ji*_^(*l*)^ is the synaptic weight from neuron *i* in layer *l* − 1 to neuron *j* in layer *l*. The synaptic weight *w*_*j*0_^(*l*)^ equals the bias, *b*_*j*_, applied to neuron *j* [34]. We used a sigmoidal logistic activation function, *g*(*v*_*j*_), to represent the nonlinear behavior between the inputs and outputs, where *v*_*j*_ is the net internal activity level of neuron *j* (i.e., the weighted sum of all synaptic inputs plus bias).

We used MLP backpropagation (MLP-BP) and MLP Scaled Conjugate Gradient (MLP-SCG) training algorithms [35]. Our custom-made software application automatically created, trained, and tested different configurations of MLPs, according to number of hidden layers and number of neurons in each hidden layer and best performance. The application begins testing the ANN with the minimum number of neurons chosen for the first hidden layer (1st hidden layer), incrementing until it reaches the maximum number of neurons (100). When this happens, a second hidden layer (2nd hidden layer) is included, first with one neuron, and a first hidden layer is set to its initial setup incrementing once again till best performance is rendered. This autonomous process is cyclically repeated with a hypothetical maximum number of neurons of 100 on 1st hidden layer and 100 on 2nd hidden layer. On each training cycle, the performance of each neural network is evaluated and stored. The autonomous creation of networks, MLP-BP or MLP-SCG, was tried with different error functions (*Mean Absolute Error* (MAE),* Mean Squared Error* (MSE),* Sum Absolute Error* (SAE), and* Sum-Squared Error* (SSE)), until best performance was reached. In the training process, the best performance is measured by two parameters that control the terminus of the training: the number of error checks and the error gradient. The latter is associated with the training performance: the lower its value, the better the training performance; and the first is incremented each time the error value in the validation set rises. These parameters were defined through an initial test with a limited number of neurons in each layer where the performance was evaluated with different gradient and error check values.

### 3.3. Radial Basis Function Neural Networks (RBNs)

RBNs are a subtype of an artificial neural network that uses radial basis functions as activation functions [37]. They consist of three layers: an input layer, a hidden radial basis neuron layer, and a linear neuron output layer. The output units implement a weighted sum of hidden-unit outputs. In RBNs, the transformation from the input space to the hidden-unit space is nonlinear whereas the transformation from the hidden-unit space to the output space is linear. When an input vector **x** is presented to such a network, each neuron's output in the hidden layer is defined by

(16) $$\begin{array}{c} \varphi_{i}\left(\;x\;\right)=e^{{-\left\|x-c_{i}\right\|}^{2}/2\sigma_{i}^{2}}, \end{array}$$

where **c**_*i*_ = [*c*_*i*1_, *c*_*i*2_,…,*c*_*im*_]  ^*T*^ ∈ *ℝ* ^*m*^ is the center vector of neuron *i* and *σ*_*i*_ is the width of the *i*th node. The response of each neuron in the output layer is computed according to

(17) $$\begin{array}{c} y_{j}=\overset{N}{\underset{i=1}{\sum }}w_{ij}\varphi_{i}\left(\;x\;\right)+b_{j}\;\text{with }j=1,2, \end{array}$$

where *w*_*ij*_ represents the synaptic weight between neuron *i* in the hidden layer and neuron *j* in the output layer and *b*_*j*_ represents the bias applied to the output neuron *j*. For more details, refer to the relevant MATLAB Documentation.

In the training process, in each iteration, two parameters were changed: the Sum-Squared Error goal and the spread value (or neuron radius) until a designated maximum value is achieved (the error goal from 1*e* − 8 to 1 and the spread value from 0.01 to 10).

### 3.4. Deep Belief Networks (DBNs)

A DBN is a generative graphical model with many layers of hidden causal variables along with a greedy layer-wise unsupervised learning algorithm. These networks are built in two separate stages. In the first stage, the DBN is formed by a number of layers of Restricted Boltzmann Machines (RBMs), which are trained in a greedy layer-wise fashion. In order to use the DBN for classification, the second stage uses the synaptic weights obtained in the RBM stage to train the whole model, in a supervised way, as a feed-forward-backpropagation neural network. For the implementation of DBNs, we used a MATLAB Toolbox developed by Palm [38].

RBMs have binary-valued hidden and visible units. If one defines the visible input layer as **x**, the hidden layer as **h**, and weights between them as **W**, the model that defines the probability distribution according to [39] is

(18) $$\begin{array}{c} P\left(\;x,h\;\right)=\frac{e^{-E\left(x,h\right)}}{Z\left(x,h\right)}, \end{array}$$

where *Z*(**x**, **h**) is a partition function given by summing over all possible pairs of visible and hidden vectors:

(19) $$\begin{array}{c} Z\left(\;x,h\;\right)=\underset{x,h}{\sum }e^{-E\left(x,h\right)}. \end{array}$$

*E*(**x**, **h**) is the energy function, analogous to the one used on a Hopfield network [40], defined as

(20) $$\begin{array}{c} E\left(\;x,h\;\right)=-\underset{i\in \text{visible}}{\sum }b_{i}^{\left(1\right)}x_{i}-\underset{j\in \text{hidden}}{\sum }b_{j}^{\left(2\right)}h_{j}-\underset{i,j}{\sum }x_{i}h_{j}w_{ij}, \end{array}$$

where *x*_*i*_ and *h*_*j*_ are the binary states of visible unit *i* and hidden unit *j* and *b*_*i*_^(1)^ and *b*_*j*_^(2)^ are the bias values of the visible and hidden layer units, respectively.

Taking into account (18) and given that there are no direct connections between hidden units in a RBM, the probability of a single neuron state in the hidden layer, *h*_*j*_, to be set to one, given the visible vector **x**, can be defined as

(21) $$\begin{array}{c} P\left(\;h_{j}=1\;|\;x\;\right)=\frac{1}{1+e^{-\left(b_{j}^{\left(2\right)}+\sum_{i}x_{i}w_{ij}\right)}}. \end{array}$$

In the same way, one can infer the probability of a single neuron, *x*_*i*_, in the visible layer, binary state being set to one given the hidden vector **h**:

(22) $$\begin{array}{c} P\left(\;x_{i}=1\;|\;h\;\right)=\frac{1}{1+e^{-\left(b_{i}^{\left(1\right)}+\sum_{j}x_{j}w_{ij}\right)}}. \end{array}$$

In the training process the goal is to maximize the log probability of the training data or minimize the negative log probability of the training data.

Palm's algorithm [38], instead of initializing the model at some arbitrary state and iterating it *n* times, initializes it with contrastive divergence algorithm introduced in [39]. For computational efficiency reasons, this training algorithm uses stochastic gradient descent instead of a batch update rule. The Restricted Boltzmann Machines (RBM) learning algorithm, as defined in [38], can be seen as follows (see [39]):

for all training samples as *t* do
*x* ^(0)^ ← *t*
*h* ^(0)^ ← sigm⁡(*x* ^(0)^ *W* + *c*) > rand()
*x* ^(1)^ ← sigm⁡(*h* ^(0)^ *W* + *B*) > rand()
*h* ^(1)^ ← sigm⁡(*x* ^(1)^ *W* + *c*) > rand()
*W* ← *W* + *α* sigm⁡(*x* ^(0)^ *h* ^(0)^ − *x* ^(1)^ *h* ^(1)^)
*b* ← *b* + *α*(*x* ^(0)^ − *x* ^(1)^)
*c* ← *c* + *α*(*h* ^(0)^ − *h* ^(1)^)
end for

where *α* is a learning rate and rand() produces random uniform numbers between 0 and 1.

Our algorithm, which uses the implementation above, trains several DBNs consecutively, varying the number of hidden neurons of the RBM and of the feed-forward neural network to a maximum of 100 hidden neurons in each of the two layers. Each RBM is trained in a layer-wise greedy manner with a learning rate of 1 for the duration of 100 epochs. After this training, the synaptic weights are subsequently used to initialize and train a backpropagation feed-forward neural network with optimal tangency activation function (11) for the hidden layers and sigmoid logistic activation function (12) for the output layer:

(23) gvi=tanh⁡vi,

(24) gvj=11+e−vj,

where *v*_*i*_, in (23), is the weighted sum of all synaptic inputs of neuron *i* plus its bias and *v*_*j*_ is the weighted sum of all synaptic inputs of the output neuron *j* plus its bias. In each training cycle the performance of each network is evaluated and stored.

### 3.5. Quantitative Measurements for Performance Evaluation

To evaluate the performance of the different classifiers we calculated accuracy, sensitivity, and specificity. A true positive (TP) was considered when the classifier output agreed with the clinical diagnosis of AD. A true negative (TN) was considered when the classifier output correctly excluded AD. Meanwhile, a false positive (FP) indicated that the classifier output incorrectly classified a healthy person with AD. The last case was a false negative (FN) when the classifier output missed AD and incorrectly classified an AD patient as a healthy person. Classification accuracy is calculated as follows:

(25) $$\begin{array}{c} \text{Accuracy}=\frac{\text{TP}+\text{TN}}{\text{TCT}}, \end{array}$$

where TCT( = TP + TN + FP + FN) is the total number of classification tests.

Sensitivity (true positive rate) and specificity (true negative rate) are calculated as follows:

(26) Sensitivity=TPTP+FN,Specificity=TNTN+FP.

## 4. Results

In this work, classifiers' performance is evaluated using the quantitative measures presented in Section 3.5.

### 4.1. Rank of Variables

**Figure 4:**
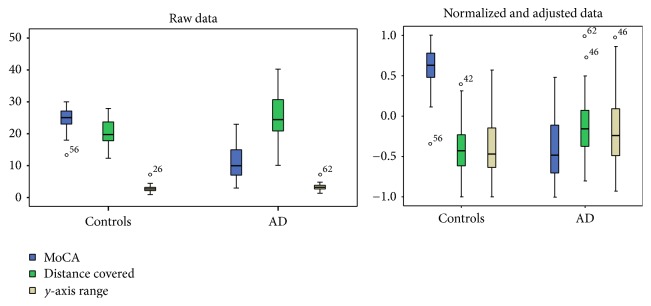
Box-plot representation of raw versus adjusted and normalized data of MoCA, total distance covered, and range of sway on *y*-axis in controls and Alzheimer's disease (AD) groups. There were significant differences between the two groups in raw data (MoCA, *p* < 0.001; distance covered, *p* < 0.001; *y*-axis range, *p* = 0.022) and after data was adjusted for age, education, height, and weight, followed by a normalization process (MoCA, *p* < 0.001; distance covered, *p* = 0.002, *y*-axis range, *p* = 0.05). Despite these statistical differences, especially in unadjusted raw data, it is important to note the significant overlap between the different individuals in the kinematic postural variables, highlighting the challenge of classification based on machine learning.

Based on the methodology described in Section 2.6, variables found with significance level below 0.05 are ranked as shown in Table 1. In this step only the training data was used to assert the statistical significance of each feature. A box-plot is presented in Figure 4 in order to show that even when data was normalized and adjusted for biometric data, with the exception of MoCA score, there is substantial overlap. This highlights the challenge for disease classification based on machine learning. In order to evaluate the rank reliability of the features, which might be dependent on the dataset size, a test was conducted by randomly reducing the dataset to 80% and 50% of its original size. The rank of variables was then conducted in 100 random repetitions and the ranks were summed and averaged to get the rank expectation. The final rank was calculated by sorting the rank expectation of all features from low to high and the top three variables were recorded and shown in Table 2, demonstrating that the rank does not change even when the dataset size is reduced 80% and 50%. This is a good indication that the set of variables found and used in this study is reliable, reproducible, and statistically meaningful even under a smaller subset of the data.

### 4.2. Error Incremental Analysis Results

**Figure 5:**
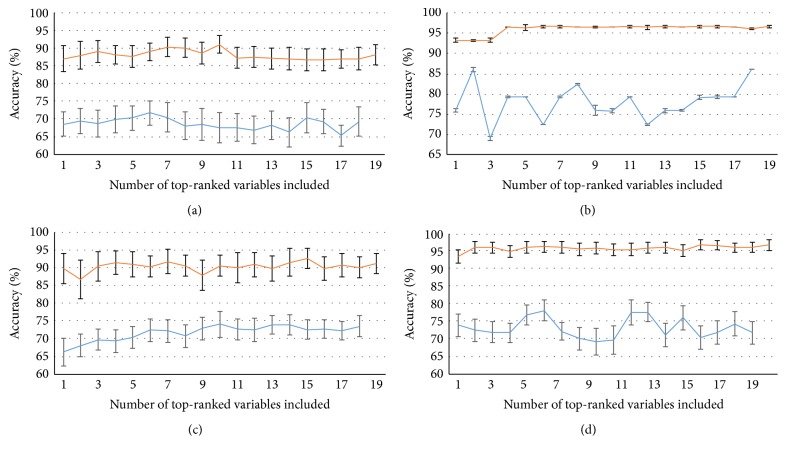
Incremental error analysis performance of accuracy with standard deviation indicated as error bar for SVM (a), MLP-BP (b), RBN (c), and DBN (d). The orange curve represents the datasets with the MoCA variable and the blue curve the datasets with only kinematic variables.

**Figure 6:**
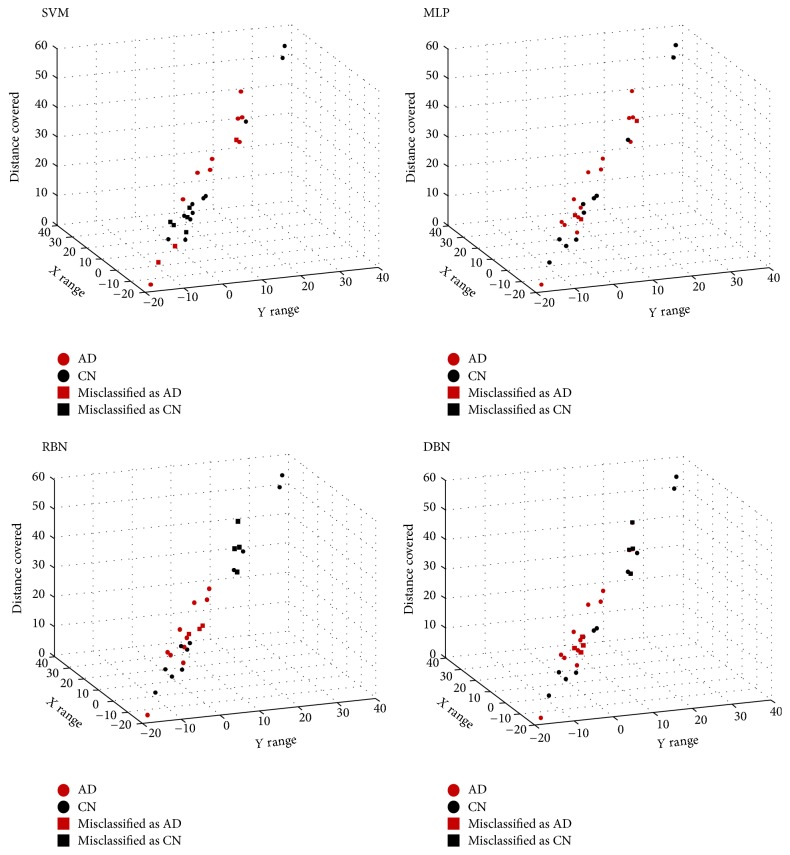
Four specific cases displaying the distribution of the subject population of the testing sets of Support Vector Machines (SVMs), Multiple Layer Perceptrons (MLPs), Radial Basis Function Neural Networks (RBNs), and Deep Belief Networks (DBNs) in the context of the top three features. This is a typical case of classification approach for Alzheimer's disease (AD) versus controls (CN).

**Figure 7:**
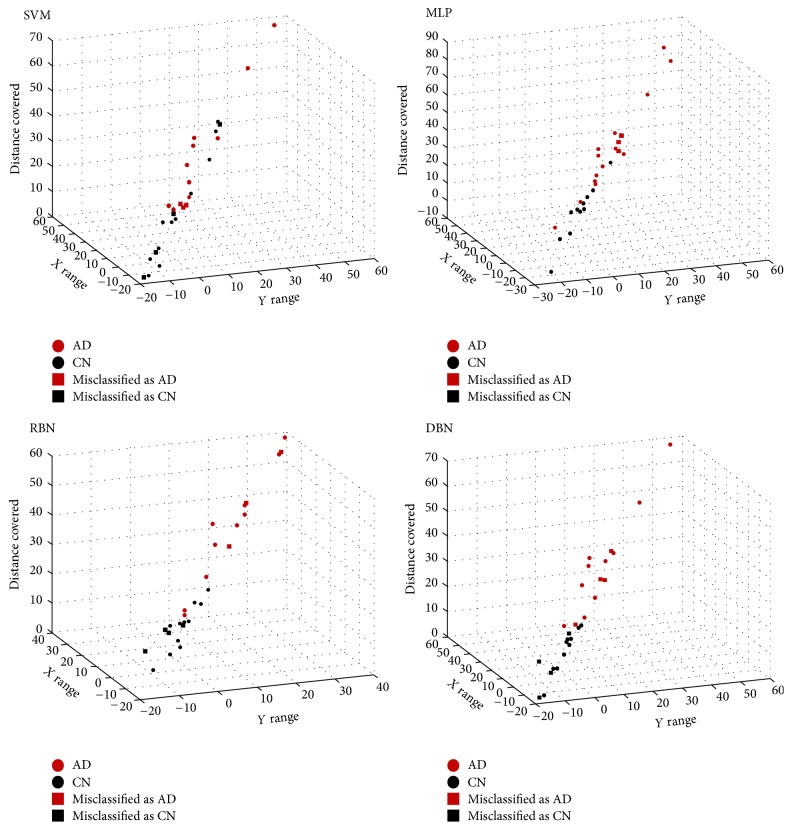
One specific case displaying the distribution of the same subject population of the testing of Support Vector Machines (SVMs), Multiple Layer Perceptrons (MLPs), Radial Basis Function Neural Networks (RBNs), and Deep Belief Networks (DBNs) in the context of the top three features. This is a typical case of classification approach for Alzheimer's disease (AD) versus controls (CN).

The objective of the incremental error analysis is to determine the number of top-ranked variables one should use in order to produce the best classification results. As in [10], the classification of AD was performed starting from the top-ranked variable and incrementally adding the next best ranked variable until all significant variables were included. The results are depicted in Figure 5. In this step the test dataset was used to estimate the accuracy values. As one can observe, all the classifiers benefited from the addition of kinematic variables, having increasingly higher accuracy values until the maximum accuracy values were achieved. These values are displayed in Table 3. Figures 6 and 7 allow inferring that AD and CN groups are generally separable as they tend to form two distinct clusters.

### 4.3. General Classification Performance

Overall, MLP achieved the highest scores with accuracy ranging from 86.1%, without MoCA, to 96.6% when kinematic postural variables were combined with MoCA. When trained with datasets combining the MoCA variable and kinematic variables, all machine-learning models showed a good classification performance, with superiority for MLP (achieving accuracy of 96.6%), followed by DBN (accuracy of 96.5%), RBN (accuracy of 92.5%), and SVM classifiers (accuracy of 91%). MLP also achieved higher sensitivity, which is also beneficial reducing the cost of misdiagnosing an AD patient as a healthy subject [41, 42]. MLP was also less susceptible to the different training iterations presenting lower standard deviation when compared to the other classifiers. Withdrawing the MoCA variable, the machine-learning classifiers also displayed a reasonably good accuracy, with results above 71%, with MLP achieving an 86.1% of accuracy rating. These results were followed by the DBN classifier with 78% accuracy rating the RBN model with 74% accuracy and lastly the SVM model achieving 71.7% accuracy rating.

## 5. Discussion

Postural control and sensory organization are known to be critical for moving safely and adapting to the environment. The investigation of postural stability under dynamic conditions, either continuous or on predictable perturbations of the supporting platform, has been used to study the complexity of balance process, which coordinates visual, vestibular, proprioceptive, auditory, and motor systems information [24, 27]. Visual suppression makes the human body more dependent on vestibular and proprioceptive systems, consequently increasing postural sway [27. Moreover, there is growing evidence that executive function and attention have an important role in the control of balance during standing and walking, as other higher cognitive processing shares brain resources with postural control [43]. Thereby, individuals who have limited cognitive processing due to neurological impairments, such as in AD, when using more of their available cognitive resources on postural control, may inadvertently increase their susceptibility to falls [44].

Having the above in context, it is not surprising that hundreds of kinematic parameters can be extracted from the IMU and each parameter can individually or in correlation represent postural body sway. While the discriminatory role of each kinematic postural variable per se is not clear, the rationale in our study was to use different and increasing difficulty balance tasks (manipulating vision and inclination), so as to increase discriminative kinematic information.

In our study we have shown that there is high intercorrelation between the different proposed kinematic variables, and even when data was normalized and adjusted for biometric characteristics, there is substantial overlap between healthy subjects and AD patients (Figures 2 and 4), which highlighted the challenge and added value on using machine-learning classifiers. As the problem being handled in this study is a classification problem, three important questions have arisen, the sample size, the number of variables per patient, and which variables compose the ideal dataset that yields the best accuracy. A small size sample has been proved to limit the performance of machine-learning accuracy [45, 46]. Also, too many variables relative to the number of patients potentially leads to overfitting, a consequence of the classifier learning with the data instead of learning the trend that underlies the data [47]. As a rule of thumb, more than 10 “events” are needed for each attribute to result in a classifier with reasonable predictive value [48]. Ideally, similar numbers of “healthy” and “unhealthy” subjects would be used in a training set, resulting in a training set that is more than 20 times the number of attributes. Since most medical studies typically involve a small number of subjects and there are essentially unlimited numbers of parameters that can be used, the possibility of overfitting has to be acquainted [49]. On one neuroimaging study, with a relatively small sample, 14 AD patients versus 20 healthy subjects, SVM reached a discriminating power of 88.2% [50]. In another study a combined approach of a genetic algorithm with ANN on EEG and neuropsychological examination of 43 AD patients versus 5 healthy subjects returned an accuracy of 85% [51].

Feature reduction can be a viable solution to tackle this problem. Besides speeding up the process of classification, it also reduces the required sizes of the training sets, therefore avoiding overfitting. Moreover, it is a way to avoid the so-called curse of dimensionality, which is the difficulty for the classifiers to learn effective models in spaces of high-dimensionality (many features) when the number of samples is limited. High dimensionality leads to overparameterization (the complexity is too high to identify all the coefficients of the model) or to poor performance of the classifiers [52]. Feature reduction can be accomplished by combining linear with nonlinear statistical analyses and/or by reducing the number of attributes. In this regard, it may contribute to simplifying the medical interpretation of the machine-learning process, by directing attention to practical clinically relevant attributes. However, choosing attributes in this retrospective manner introduces a post hoc subjective element into analyses [53]. Previous works have shown that feature reduction/selection methods have a positive effect on the classifiers' performance [10, 54–57].

Using the error incremental analysis method one was able to determine the optimal decisional space in which the classification of AD versus controls is carried out. By testing the variable's ranks with reduced datasets (80% and 50%) one verified that the rank of the top variables did not change, indicating that the combination of features suggested in this study is reliable and statistically relevant. As also indicated in [10], it can be argued that incremental error analysis does not cover all the possible combinations of features. Assessing all the combinations of variables, besides being exhausting and extremely time-consuming, is unnecessary due to the ranking of variables done in the beginning of the study.

Several biomarkers, such as demographic, neuropsychological assessment, MRI imaging, and genetic and fluid biomarkers, have been used in the diagnosis of AD [58]. Even though neuroimaging biomarkers, such as normalized hippocampus volume, have reached high accuracy rates in the diagnosis of AD, they are a structural anatomical evaluation of the brain and not its function. Patients with higher cognitive reserve, due to education and occupational attainment, can compensate their deficits and be more resilient to structural pathological brain changes [59]. As such, neuropsychological test, a functional cognitive assessment, can outperform MRI imaging, in the diagnosis of AD or even in the differential diagnosis with other dementias [60]. In our study, in general, all classifiers—SVM, MLP, RBN, and DBN—have presented very satisfying results: MLP classifier model had the highest performances, being more consistent between the different training iterations. As expected, adding MoCA scores yielded higher accuracy rates, with above 90% accuracy rates for all classifiers. As the diagnosis of AD is supported on cognitive evaluation, including the MoCA evaluation score into the dimensional space has to be considered with caution, as it can result in biased accuracy estimates. Nevertheless, relying solely on kinematic data, we achieved performance rates ranging from 71.7 to 86.1%. Interestingly, our results are in contrast to other studies where the combination of biomarkers, MRI imaging and neuropsychological assessment, had a detrimental effect of classification accuracy rates, probably as a consequence of redundancy between these variables that represent the same dysfunction [60]. Even though further studies are needed to elucidate the correlation between postural control and cognition, we have shown that the combination of neuropsychological assessment and postural control analysis are complementary in the diagnosis of AD. Our results are consistent with other studies where performances within 88% [6, 61] and 92% [12] were achieved using neural networks. DBNs have also displayed very good performances, which are compatible to the performance records of classifying AD based on neuroimaging data [14, 41, 62]. SVM is considered useful for handling high-dimensional data [53], as it efficiently deals with a very large number of features due to the exploitation of kernel functions. This is particularly useful in applications where the number of attributes is much larger than the number of training objects [63]. However, we did not find SVM to be the superior classifier. A drawback of SVM is that the problem complexity is not of the order of the dimension of the samples, but of the order of the samples.

## 6. Conclusion

Our work shows that postural kinematic analysis has the potential to be used as complementary biomarker in the diagnosis of AD. Machine-learning classification systems can be a helpful tool for the diagnosis of AD, based on postural kinematics, age, height, weight, education, and MoCA. We have shown that MLPs, followed by DBN, RBN, and SVM, are useful statistical tools for pattern recognition on clinical data and neuropsychological and kinematic postural evaluation. Specifically, in the datasets relying solely on kinematic postural data (i) MLP achieved a diagnostic accuracy of 86% (sensitivity: 79%; specificity: 93%); (ii) DBN achieved a diagnostic accuracy of 78% (sensitivity: 79%; specificity: 77%); (iii) RBN achieved a diagnostic accuracy of 74% (sensitivity: 71.3%; specificity: 76.7%); and finally (iv) SVM achieved a diagnostic accuracy of 71.7% (sensitivity: 65%; specificity: 78.4%).

These results are competitive in comparison to results reported in other recent studies that make use of other types of data, such as MRI, PET, EEG, and other biomarkers (see [10] for a list of performances). These results are also competitive when compared to [10], which also used a neuropsychological variable (minimental state examination) (MMSE) in combination with MRI, obtaining results of 78.2% and 92.4% accuracy when the SVM is trained with datasets without and with the MMSE variable, respectively. Crossing a statistical model (nonparametric Mann–Whitney* U* test) to reduce the number of input variables with machine-learning models has proved to be an advantageous preprocessing tool to a certain extent. This is corroborated by observing that the best results were obtained by the classifiers when trained with reduced datasets.

Future perspectives of our work are to collect a larger dataset of AD patients and healthy subjects, so as to better comprehend the discriminatory role of each kinematic postural variable per se as well as its interdynamic interaction, in the process of maintaining balance within the limits of stability. Other future step, would be to evolve from a nonstatic to a dynamic paradigm, that is to say, simultaneously studying the constant dynamics of postural control and cognition (e.g., attention) on nonstationary increasingly difficult levels of balance and cognition tasks.

## Figures and Tables

**Table 1:** Ranking of the 19 variables in the input vector, Alzheimer's disease versus controls groups.

Variable	Rank
MoCA	1
Distance covered	2
*Y* range	3
*X* range	4
Maximum distance	5
*Y* maximum	6
Maximum Pitch	7
Mean distance	8
*Y* mean	9
*X* maximum	10
Radius of dispersion	11
Mean velocity	12
*X* mean	13
Maximum Roll	14
Mean pitch angle	15
Maximum velocity	16
Mean roll angle	17
Minimum Roll	18
Minimum Pitch	19

**Table 2:** Summary of top-ranked variables with varying dataset size.

Dataset size	Rank of variables
100%	MoCA Distance covered *Y* range
80%	MoCA Distance covered *Y* range
50%	MoCA Distance covered *Y* range

**Table 3:** Best results obtained with each classifier trained with datasets with and without the MoCA variable. Between parentheses are the minimum and maximum values obtained. All results are in percentage.

	Accuracy	Sensitivity	Specificity	Best decisional space
SVM with MoCA	91 (75–96.4)	89.3 (64.3–100)	92.7 (71.4–100)	Top 10 features
SVM without MoCA	71,7 (53.6–92.9)	65 (35.7–92.9)	78.4 (35.7–100)	
MLP with MoCA	96.6 (96.5–100)	100 (100–100)	94.9 (94.7–100)	Top 11 features and top 15 features
MLP without MoCA	86,1 (79.3–86.2)	78.5 (77.8–78.6)	93.1 (81.8–93.3)	
RBN with MoCA	92,5 (75–100)	90.4 (71.4–100)	94.5 (78.6–100)	Top 15 features
RBN without MoCA	74,0 (53.6–82.1)	71.3 (50–100)	76.7 (42.9–100)	
DBN with MoCA	96,5 (89.3–100)	95.3 (85.7–100)	97.7 (85.7–100)	Top 15 features
DBN without MoCA	78,0 (57.1–92.9)	79 (14.3–100)	77 (21.4–100)	
